# An unusual case of diffuse large B‐cell lymphoma complicating a Crohn's disease

**DOI:** 10.1002/ccr3.3364

**Published:** 2020-09-17

**Authors:** Meriam Sabbah, Fatma Ben Farhat, Nawel Bellil, Fatma Khanchel, Asma Ouakaa, Dalila Gargouri

**Affiliations:** ^1^ Department of Gastroenterology Habib Thameur Hospital Tunis Tunisia; ^2^ Faculty of Medicine of Tunis University of Tunis El Manar Tunis Tunisia; ^3^ Department of Pathology Habib Thameur Hospital Tunis Tunisia

**Keywords:** Crohns disease, gastroenterology and hepatology, lymphoma, oncology

## Abstract

Clinicians should keep in mind that Crohn's disease may be complicated by neoplasia such as adenocarcinomas or lymphomas, especially if the disease duration is long and the patient is under immunosuppressive therapy.

## Graphical abstract

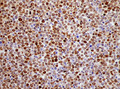

## 1 INTRODUCTION

An increased incidence of non‐Hodgkin's lymphoma has been reported in patients with inflammatory bowel disease, particularly in those receiving immunosuppressive therapy. This complication generally arises in sites of active inflammatory disease. We report an unusual case of diffuse large B‐cell lymphoma complicating an ileocolic Crohn's disease, which appeared suddenly and became widespread rapidly. The diagnosis was confirmed by peritoneal cytology with immunochemistry.

The association between Crohn's disease and malignancy is well documented. The risk of developing small bowel adenocarcinoma is slightly increased in patients with Crohn's disease.^1^ However, the association between Crohn's disease and non‐Hodgkin's lymphoma remains controversial as the number of cases is not big enough to establish a definitive relationship.^2^ Intestinal lymphoma generally arises in long‐standing Crohn's disease, in locations where the inflammatory disease is highly active. In this paper, we report an unusual case of Diffuse large B‐cell lymphoma in a patient with an 18‐year history of ileocolic Crohn's disease.

## 2 CASE REPORT

A 42‐year‐old woman with an 18‐year history of Crohn's disease presented with malnutrition and abdominal pain. She had been intermittently treated with corticosteroids and azathioprine and had been hospitalized on multiple occasions for intermittent small bowel obstruction.

A colonoscopy performed four months earlier had revealed the appearance of a severe flare of ileocolic Crohn's disease with probable ileal stenosis. The histological examination had shown catarrhal colitis with signs of chronic Crohn's disease. Computed tomography (CT) with contrast revealed a symmetric circumferential thickening of the intestinal wall in the last ileal segment related to Crohn's disease.

**Figure 1:**
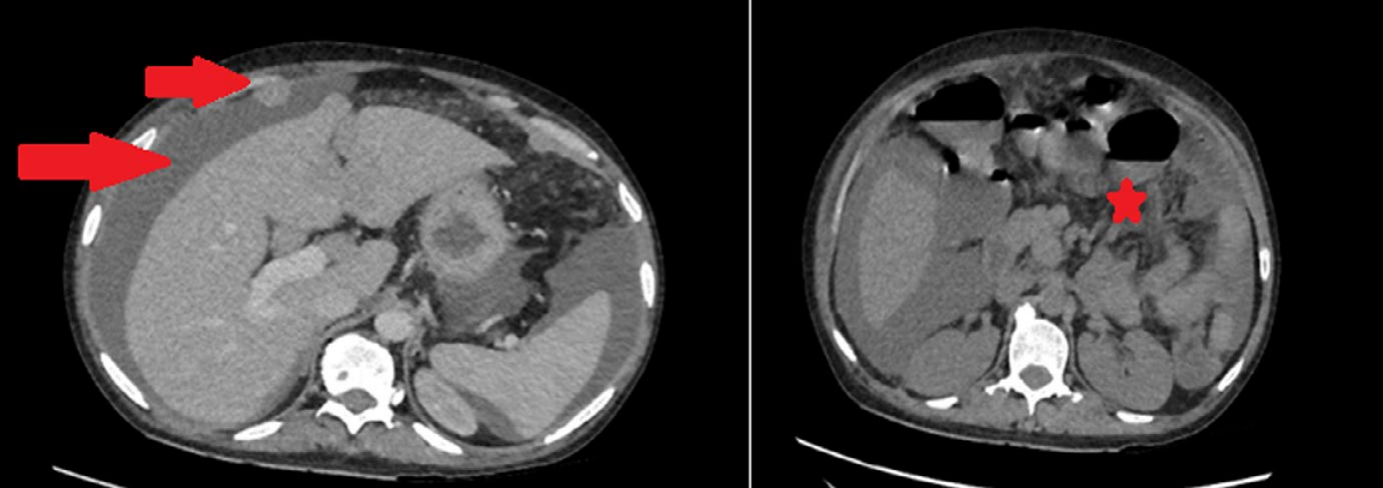
Abdominal CT scan showing irregular circumferential parietal thickening of the last ileal segment, and multiple mesenteric and abdominal lymph nodes with malignant ascites

**Figure 2:**
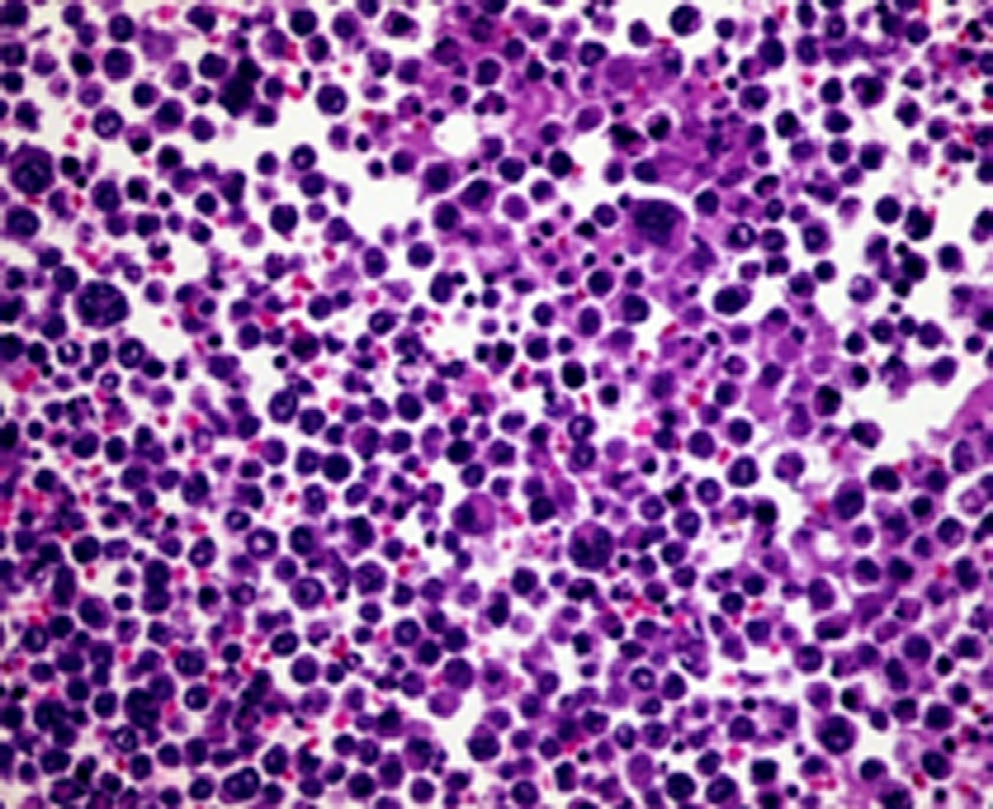
Histological examination of ascites: HE (×400) lymphoid proliferation

**Figure 3:**
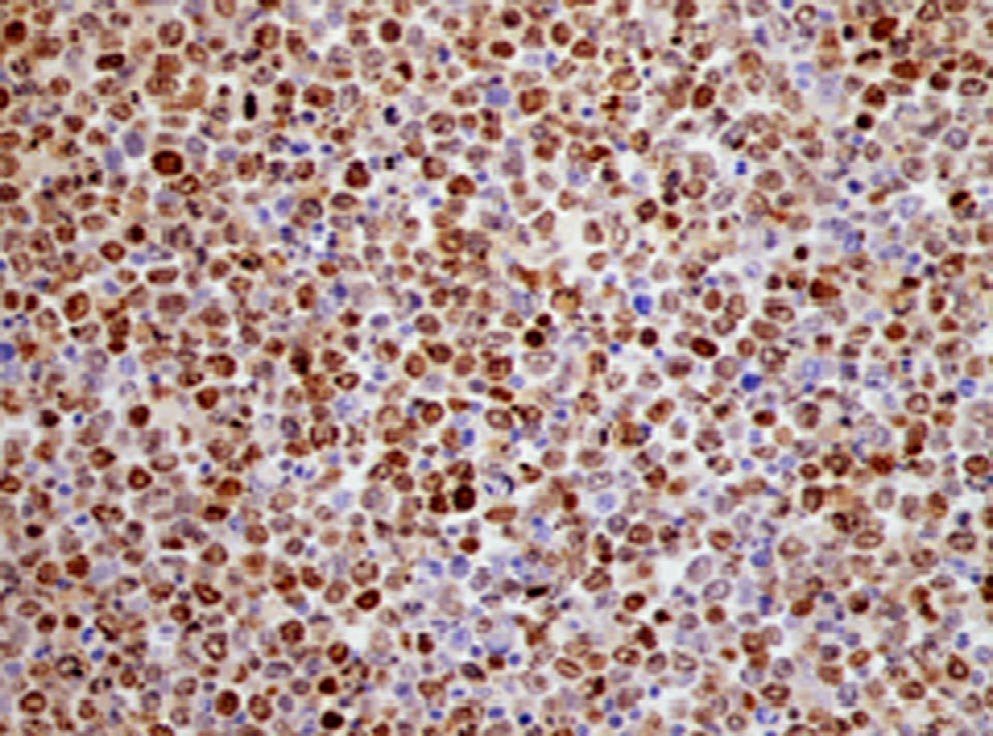
Positivity of immunostaining Ki‐67 (×400)

**Figure 4:**
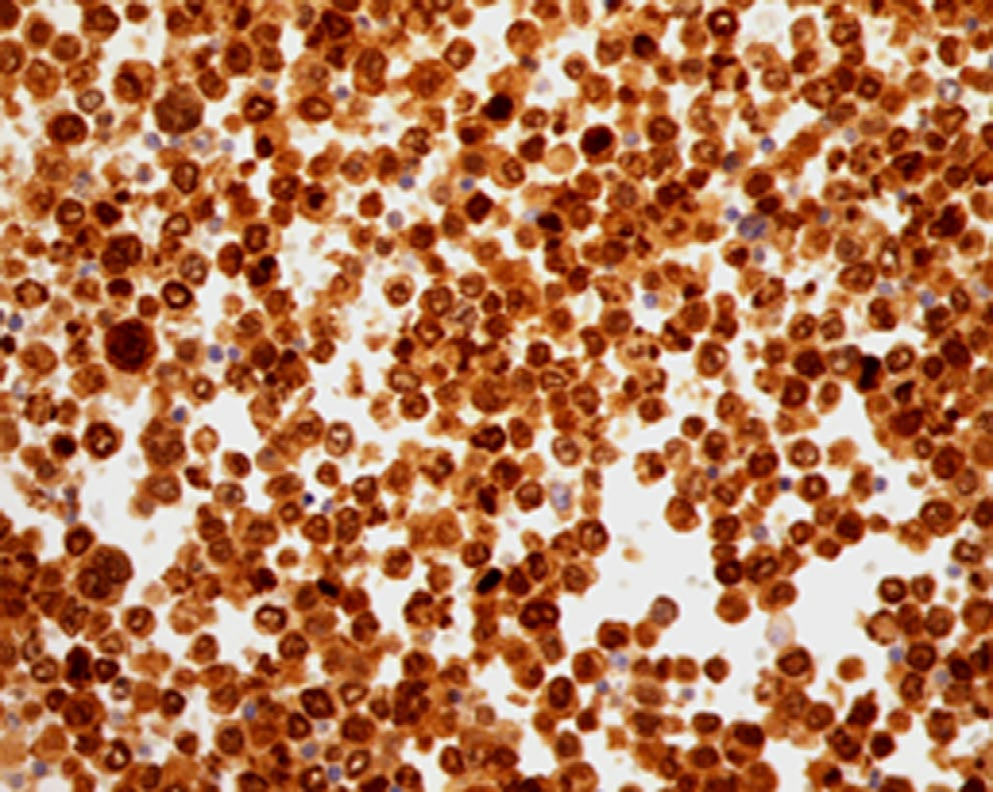
Positivity of immunostaining Mum1 (×400)

She presented to the clinics with a two‐week history of abdominal pain, vomiting, and weight loss. Upon hospital admission, her vital signs were blood pressure 130/70 mm Hg, pulse 100 beats/min, and temperature 38.6°C. Her body mass index (BMI) was calculated at 22 kg/m^2^. Abdominal examination found diffuse tenderness and distention with ascites. The laboratory data, summarized in Table 1, showed abnormally elevated inflammatory markers (CRP = 310, WBC = 11 050). An abdominal and chest contrast CT revealed an irregular, circumferential thickening of the intestinal wall in last ileal segment, multiple mesenteric and abdominal lymph nodes with malignant ascites, and multiple vertebral osteolytic lesions (Figure 1). This aspect called to mind a lymphomatous transformation with peritoneal carcinomatosis and bone metastases. Ascites cytology with immunochemistry concluded to large B‐cell lymphoma with positive CD20 (Figures 2, 3, 4). The patient developed acute kidney failure with severe metabolic acidosis. She died several days later due to pulmonary edema.

**Table 1:** Laboratory data

Laboratory tests	Day 1 of admission	Day 7
Hemoglobin (g/dL)	9.8	9.1
Platelet Count	383 000	370 000
White blood cell count	8130	11 050
Prothrombin time (%)	100	84
Asparate aminotransferase (U/L)	33	—
Alanine aminotransferase (U/I)	13	—
Total bilirubin (mg/L)	11	—
Alkaline phosphatase (U/I)	75	756
Creatinine (mg/L)	74	139
Sodium (mEq/L)	138	3.2
Potassium (mEq/L)	4.5	22
C‐reactive protein (mg/L)	310	—
Creatinine phosphokinase (UI/L)	42	—
Lactic acid dehydrogenase (UI/L)	1475	—
Corey embryonic antigen (ng/mL)	2.7	—
Albuminemia (gL)	25	—
pH	7.47	7.24
PaO_2_	80	125
PCO_2_	34	18
HCO_3_^−^	24.7	7.7

## 3 DISCUSSION

Primary malignant lymphoma of the gastrointestinal tract is rare.^3^ Most cases occur in the stomach, but sometimes the small bowel and colon are involved. Inflammatory bowel disease (IBD), especially ulcerative colitis, is known to increase the risk of adenocarcinoma which presents with extensive bowel involvement and persistent colitis.^4^ However, primary gastrointestinal lymphoma was rarely reported in association with IBD, being more common in chronic ulcerative colitis. Shepherd et al^5^ reported seven cases associated with ulcerative colitis and three cases associated with Crohn's disease.

Adrian et al^6^ reported that the risk factors for colonic lymphoma in UC were, in three principal aspects, similar to those of colitis‐related colonic adenocarcinoma: younger age at cancer onset, universal colitis, and long‐standing quiescent disease. In contrast, lymphomas in Crohn's disease occurred within 10 years of the initial diagnosis,^5^ and small bowel lymphoma was more frequent. Early diagnosis of lymphoma is challenging because of the similar radiologic appearance with Crohn's disease.^8^ Non‐Hodgkin's lymphoma is more frequent than Hodgkin's lymphoma in IBD.^7^ Tumors in Crohn's disease are aggressive since they are high grade. Diffuse large B‐cell lymphoma (DLBCL) is the most common subtype of aggressive lymphomas and has a lower survival rate than MALT lymphoma.^11^ The prognosis and management of this tumor depend on the staging. Patients at lower stages (I and II) had a longer cumulative survival than patients at stage III or IV.^12^ Containing‐strategy chemotherapy may be the optimal choice for intestinal DLBCL.^12^

Even though association between Crohn's disease and lymphoma is uncertain, some findings support its existence including the fact that the majority of reported tumors have arisen at sites of active inflammatory bowel disease (intestinal tract).^5^ A possible factor in the genesis of lymphoma is immunosuppression. In fact, a published meta‐analysis showed a fourfold increased risk of non‐Hodgkin's lymphoma in a subgroup of IBD patients treated with azathioprine and/or 6‐mercaptopurine.^9^ Other mechanisms can also explain this association including primary immunologic defects associated with IBD. Auen et al showed that certain patients with Crohn's disease may have deficits in both T‐ and B‐lymphocyte populations secondary to the high activity and chronicity of the disease.^10^ Besides, frequent exposure to X‐rays increases the risk of lymphoma.^6^ Our patient had large cell lymphoma, known to occur exclusively with immunosuppression‐induced lymphoma. In fact, she had been treated with corticosteroids and azathioprine for many years.

Some patients did not receive azathioprine or prednisone before diagnosis of the lymphoma, so these medications could not have played an essential role in the development of lymphoma.^6^

## 4 CONCLUSION

There is good evidence that malignant lymphoma of the bowel is a rare but significant complication of inflammatory bowel disease, apparently more common in chronic ulcerative colitis. Since the prognosis of lymphoma depends on the stage of the disease at the time of diagnosis, we suggest meticulous monitoring to diagnose the disease early so that treatment can be initiated promptly, which can have significant benefits.

## CONFLICTS OF INTEREST

None declared.

## AUTHOR CONTRIBUTIONS

SM and BFF: wrote the manuscript. BN and OA: held the patient in the department. KF: provided the cytology examination results and pictures. GD: reviewed the manuscript.
